# Hyper-excitability and epilepsy generated by chronic early-life stress

**DOI:** 10.1016/j.ynstr.2015.03.001

**Published:** 2015-03-24

**Authors:** Céline M. Dubé, Jenny Molet, Akanksha Singh-Taylor, Autumn Ivy, Pamela M. Maras, Tallie Z. Baram

**Affiliations:** a Department of Anatomy, Pediatrics, and Neurology, University of California-Irvine, Irvine, CA, USA; b Department of Neurobiology, Pediatrics, and Neurology, University of California-Irvine, Irvine, CA, USA

**Keywords:** Stress, Seizures, Epilepsy, Infantile spasms, Corticotropin releasing hormone, Amygdala

## Abstract

Epilepsy is more prevalent in populations with high measures of stress, but the neurobiological mechanisms are unclear. Stress is a common precipitant of seizures in individuals with epilepsy, and may provoke seizures by several mechanisms including changes in neurotransmitter and hormone levels within the brain. Importantly, stress during sensitive periods early in life contributes to ‘brain programming’, influencing neuronal function and brain networks. However, it is unclear if early-life stress influences limbic excitability and promotes epilepsy. Here we used an established, naturalistic model of chronic early-life stress (CES), and employed chronic cortical and limbic video-EEGs combined with molecular and cellular techniques to probe the contributions of stress to age-specific epilepsies and network hyperexcitability and identify the underlying mechanisms.

In control male rats, EEGs obtained throughout development were normal and no seizures were observed. EEGs demonstrated epileptic spikes and spike series in the majority of rats experiencing CES, and 57% of CES rats developed seizures: Behavioral events resembling the human age-specific epilepsy infantile spasms occurred in 11/23 (48%), accompanied by EEG spikes and/or electrodecrements, and two additional rats (9%) developed limbic seizures that involved the amygdala. Probing for stress-dependent, endogenous convulsant molecules within amygdala, we examined the expression of the pro-convulsant neuropeptide corticotropin-releasing hormone (CRH), and found a significant increase of amygdalar--but not cortical--CRH expression in adolescent CES rats.

In conclusion, CES of limited duration has long-lasting effects on brain excitability and may promote age-specific seizures and epilepsy. Whereas the mechanisms involved require further study, these findings provide important insights into environmental contributions to early-life seizures.

## 1 Introduction

Stress is a common precipitant of seizures in individuals with epilepsy (Temkin and Davis, 1984, Neugebauer et al., 1994, Frucht et al., 2000, Spector et al., 2000, Haut et al., 2007, Nakken et al., 2005), and may provoke seizures by several mechanisms including changes in neurotransmitter and hormone levels within the brain (Nakken et al., 2005, Baram, 1993, Joëls, 2009, Danzer, 2012). In addition, an age-dependent epilepsy of infants called infantile spasms (IS), responds to the stress hormones ACTH and prednisone/prednisolone (Hrachovy et al., 1983, Snead et al., 1989, Baram et al., 1996, Mackay et al., 2004). The mechanisms of the anticonvulsant effects of these hormones might involve a suppression of the endogenous proconvulsant stress peptide corticotropin-releasing hormone (CRH) (Baram, 1993, Huang, 2014, Brunson et al., 2001a, Stafstrom et al., 2011). These facts illustrate that there is a complex relationship between stress and seizures, with important clinical implications.

Whereas much remains to be learned about stress, seizures and epilepsy in the mature brain (Joëls, 2009, Danzer, 2012), even less is known about pro-convulsant and pro-epileptic effects of stress early in life (Baram, 1993, Huang, 2014, Hatalski et al., 1998, Velísek et al., 2007). There is epidemiological evidence for increased incidence of epilepsy in children growing up in presumably stressful, resource-poor environments (Shamansky and Glaser, 1979), yet the effects of early-life stress on the risk of developing epilepsy have been little studied in humans (Li et al., 2008, van Campen et al., 2012). In rodent models, stress during development is pro-convulsant in several models of seizures and epilepsy: Prenatal maternal restraint increases the severity of status epilepticus (Sadaghiani and Saboory, 2010), enhances the effects of postnatal N-methyl-d-aspartate (NMDA) receptor agonists (Velísek et al., 2007, Chachua et al., 2011), decreases afterdischarge threshold and enhances kindling rates (Edwards et al., 2002). Early postnatal stress increases seizure susceptibility in several models of seizures including amygdala kindling, freeze lesion followed by hyperthermic seizures and chemo-convulsant-induced seizures (Salzberg et al., 2007, Desgent et al., 2012, Schridde et al., 2006).

These data suggest that both prenatal and early postnatal stresses enhance vulnerability to seizures. Perinatal and early-life stresses take place during critical periods of brain development when synapses form, networks get established and environmental signals may have long-lasting effects (Bale et al., 2010). Stress may influence all of these processes and may alter brain circuits, cellular properties, and synaptic connections (Huang, 2014, Brunson et al., 2001b). These changes, in turn, may render the individual more vulnerable to seizures and epilepsy via poorly understood interactions with a number of stress mediators (Joëls, 2009, McEwen, 2007, Baram and Hatalski, 1998, Wang et al., 2001).

In addition to the effects of stress on seizure susceptibility, early-life stress might provoke the emergence of spontaneous seizures (epileptogenesis). This is an important question, because epilepsy, especially childhood-onset epilepsy, is associated with adverse consequences: children with epilepsy perform worse than expected in school, employment, marriage, and parenthood (Camfield et al., 1993, Gaitatzis et al., 2004). They have increase prevalence of depression and intellectual dysfunction (Berg et al., 2008, Cormack et al., 2007). Findings in animal models of seizures support detrimental effects of seizures on cognition and emotion (Holmes et al., 2002, Lynch et al., 2000, Dubé et al., 2009).

To examine directly whether sustained early-life stress promotes hyperexcitability and epilepsy, we used a well characterized model of chronic early-life stress (CES) (Gilles et al., 1996, Avishai-Eliner et al., 2001, Ivy et al., 2008). We then probed the mechanisms underlying these stress-provoked changes to brain-network excitability.

## 2 Material and methods

### 2.1 Animals

Subjects were progeny of timed-pregnancy Sprague–Dawley rats. Rats were maintained in quiet facilities under controlled temperatures and light–dark cycle. Cages were monitored every 12 h for the presence of pups and the date of birth was considered postnatal day (P) 0. Pups were mixed among litters, and litter size was adjusted to 12 per dam if necessary, to obviate the potential confounding effects of genetic variables and of litter size. Litters of all experimental groups contained equal numbers of males. When weaned (on P21), male rats were housed 2–3 per cage. All experiments were performed in accordance with the National Institutes of Health (NIH) guidelines on laboratory animal welfare and approved by the University of California–Irvine Institutional Animal Care and Use Committee.

### 2.2 The chronic early-life stress paradigm

CES was induced in rat pups from P2 to P9 as described previously (Gilles et al., 1996, Ivy et al., 2008, Molet et al., 2014). Stress in pups was provoked by unpredictable and fragmented nurturing behaviors of rat dams (Baram et al., 2012). These behaviors, in turn were induced by equipping the cages with limited nesting material that prevented the dam from constructing a satisfactory nest and altered her behavior (Ivy et al., 2008, Molet et al., 2014). Briefly, on P2, pups (male and female) from several litters were mixed among dams and those assigned to the CES groups were transferred to cages with limited bedding and nesting material. Specifically, cages were fitted with a plastic coated aluminum mesh platform to sit approximately 2.5 cm above the cage floor. Bedding was reduced to only cover the cage floor sparsely, and one-half of a paper towel was provided for nesting maternal. Control dams and pups resided in bedded cages, containing ∼0.33 cubic feet of sanitary chips. Control and experimental cages were undisturbed during P2–P9. Maternal nursing behaviors were monitored during the week of CES as described (Molet et al., 2014). At P21, pups were weaned, and male pups were used for the experiments.

### 2.3 Electrode implantation

Animals (*n* = 36, 23 CES and 13 controls) underwent surgery at P11 – P15. Two EEG systems were used: a tethered and a telemetric. For the tethered setup, a first rat cohort (6 CES and 4 control) was implanted with bipolar stainless steel electrodes (Plastics One, Roanoke, VA) in both hippocampi (coordinates: AP −2.0, L 1.8, V −2.6 mm with reference to Bregma) (Paxinos and Watson, 1998), and with 7 dural screw-electrodes over the parietal frontal and temporal cortices. A second cohort of rats (11 CES and 5 control) was implanted bilaterally with bipolar electrodes in amygdala (coordinates: AP -1.6, L 3.6, V −8.4 mm with reference to Bregma) as well as with one electrode over the right fronto-parietal cortex. The amygdala electrodes (twisted wires) were connected in a sequential manner in a bipolar montage. The electrode going to grid 2 of the first derivation was also connected to grid 1 of the next derivation.

To record from the surface of the cortex, stainless steel insulated electrodes (E363) were used and consisted of electrodes with mounting screw and socket contact. A surface electrode positioned over the cerebellum was used as a ground electrode for all electrode assemblies used here. The intracranial electrodes used in this study (Plastic One; E363/2-2 TW) were made of a stainless steel wire (bare diameter 200 μm) insulated with polyimide (electrode diameter insulated 230 μm) and ending with a female socket contact. The electrodes were supplied twisted together, i.e., bipolar electrodes. The insulation at the tip level was removed for the terminal 0.1 mm, and tips separated by 1 mm. Screws were placed over the cortex and dental acrylic was used to anchor the electrodes to the pedestal. We recorded potentials either between two cortical electrodes, one in the left and one in the right fronto-parietal cortices; or between one of the twisted electrodes in the right amygdala and the right cortical electrode. The electrodes were connected through a pedestal to EEG leads (6 channel electrode cable with 363 plug; Plastics One Roanoke, VA).

For the telemetry system, a third rat cohort (6 CES and 4 controls) was implanted with bipolar electrodes in the right amygdala and with two dural electrodes over the right and left fronto-parietal cortices. The telemetry transmitter allows simultaneous recordings of two channels. Each channel received input from the 2 wires of one intra-amygdala bipolar electrode. The radiotelemetry unit (the two-channel PhysioTel Implantable Transmitter F20-EET; Data Sciences International [DSI], St Paul, MN, USA) was positioned in a pocket created subcutaneously in the rat flank through a scalp incision. All electrodes were fixed in place with dental acrylic and connected to the leads of the radiotelemetry unit, and then covered and fixed to the skull with dental acrylic cement.

For all rats, electrode placement was verified post hoc (Choy et al., 2014), and they were all located in amygdala or hippocampus as intended. Because of the size of the amygdaloid complex in neonatal rats, we can comfortably state only that all electrode tips were in the central nucleus or medial portion of the basolateral nucleus.

### 2.4 Long-term digital video-EEG recordings

After a day of recovery, experimental and control rats were recorded via one of the two systems described above: The tethered system employed bio-amplifiers using Powerlab 8SP (AD Instruments, Grand Junction, CO) equipped with Chart 4 for Windows. This software was used to acquire the data, with band-pass frequency filters of 0.1 and 200 Hz, a notch filter at 60 Hz, and a sampling rate of 400 Hz. Video was acquired using a commercial webcam (Logitech Quickcam, Ultra Vision; Logitech International) that was synchronized with the EEG. The telemetric system employed the Dataquest A.R.T. acquisition system (DSI). The transmitters broadcasted digitized data via radio frequency signals to PhysioTel Receivers (RPC-1). The receivers converted the telemetry information to a form readily accessible by DSI's Dataquest platform. The sampling rate was 100 Hz and the video was captured using four analog cameras (Samsung SDN-550N) connected to a network video server (Axis Q7404 4 channels video encoder) synchronized to the EEGs.

Digital video EEG recordings were conducted for 2–4 weeks as described previously (Dubé et al., 2010, Choy et al., 2014). To avoid maternal rejection and inanition or cannibalism, pups were recorded intermittently prior to weaning. When using the tethered system, animals were recorded for an hour a day, and the order of recording was rotated to avoid potential diurnal variability in brain excitability. When employing the telemetry system, rats were recorded for two hours a day until P21. After weaning, continuous digital video EEG recordings were conducted.

### 2.5 Video and EEG review and analysis

Investigators unaware of the experimental group-status of each rat-EEG first analyzed the EEGs visually, scanning for seizures and for interictal activity and excluding potential motion artifacts (56). The concurrent video-recordings were analyzed for behavioral epileptic manifestations. To classify an event as a potential seizure, both EEG- and behavioral phenomena were required. Electrographically, seizures were defined as events consisting of spikes (inflections characterized by duration of <50 mSec and amplitude > 2 fold background) that lasted more than 6 s. Whereas there are numerous operational definitions of seizures, none is satisfactory, and we chose 6 s as a minimum duration because this is twice the reported duration required for alteration of consciousness in absence seizures in children, and because it is conventional in rodent models (Dubé et al., 2010, Choy et al., 2014, Nairismägi et al., 2004, Dubé et al., 2006). Additionally, the EEGs were analyzed using the seizure detection module of the Neuroscore software (DSI).

### 2.6 *In situ* hybridization histochemistry (ISH) for CRH mRNA

*In situ* hybridization histochemistry was performed on a separate cohort consisting of CES and control rats that were sacrificed on P19. The ISH method has been described in detail previously (Avishai-Eliner et al., 2001, Ivy et al., 2008). Briefly, 20 μm coronal sections were collected on gelatin-coated slides and stored at −80 °C. Sections were thawed, air dried, fixed in paraformaldehyde, dehydrated, and rehydrated through graded ethanols, then exposed to 0.25% acetic anhydride in 0.1 M triethanolamine (pH 8) and dehydrated. Prehybridization and hybridization steps were performed at 40 °C in a humidified chamber. Following one hour of prehybridization, sections were hybridized overnight (20 h) with a deoxyoligonucleotide probe complementary to the coding region of CRH mRNA and 3′-end-labeled with ^35^S-dATP. Sections were then washed and apposed to film (Hyperfilm β-Max; Amersham, Arlington Heights, IL) for 7–14 days.

### 2.7 CRH immunocytochemistry

Immunocytochemistry (ICC) was performed on a separate cohort of rats (3 control and 3 CES). Briefly, juvenile rats (P45) were euthanized with sodium pentobarbital and perfused with fresh 4% paraformaldehyde in 0.1 M sodium phosphate buffer (PB; pH 7.4, 4 °C). Brains were cryoprotected and stored, then sectioned coronally into 20 μm thick slices using a cryostat. CRH ICC was performed on free-floating sections as previously described (Chen et al., 2001). Briefly, after washing (3 × 5 min) with 0.01 m PBS containing 0.3% Triton X-100 (PBS-T; pH 7.4), sections were treated for 30 min in 0.3% H_2_O_2_/PBS, followed by blockade of nonspecific sites with 5% normal goat serum in PBS for 30 min. After rinsing, sections were incubated for 2 d at 4 °C with rabbit anti-CRH antiserum (1:20,000; a gift from Dr. W. W. Vale, Salk Institute) in PBS containing 1% bovine serum albumin, and washed in PBS-T. Sections were incubated in biotinylated goat-anti-rabbit IgG (1:200; Vector Laboratories) in PBS for 2 h at room temperature. After washing, sections were incubated in the avidin–biotin–peroxidase complex (ABC) solution (1:100; Vector Laboratories) for 2 h and rinsed (3 × 5 min PBS-T), and the reaction product was visualized by incubating the sections in 0.04% 3,3′-diaminobenzidine (DAB) containing 0.01% H_2_O_2_. Sections were mounted on poly-l-lysine-coated slides and coverslipped with Permount (Fisher Scientific).

### 2.8 RNA isolation and quantitative reverse transcription PCR (qRT-PCR)

The amygdalae were dissected using pre-chilled RNase free instruments under a light microscope, and processed immediately. Total RNA was isolated from the tissue using the RNeasy mini kit (Qiagen) as per manufacturer's protocol. RNA purity and quantity was determined using a nanodrop (Thermo Scientific). 1 μg of RNA was converted to cDNA with random hexamers using transcriptor first strand cDNA synthesis kit following manufacturer's protocol (Roche). Sybr Green PCR analysis was performed using cDNA samples in triplicate on a Roche Lightcycler 96 system (Roche) for CRH and GAPDH transcripts. GAPDH served as the internal control, and relative quantification of mRNA expression was determined using the cycle threshold method (2ˆ-ΔΔCt). Minus-reverse transcription and non-template controls were used to eliminate the possibility of genomic contamination or false positive analyses.

Primer Sequences were: CRH: (fwd: 5′- GAAACTCAGAGCCCAAGTACGTTGAG -3’; rev: 5′- GTTGTTCTGCGAGGTACCTCTCTCAG -3′).

GAPDH: (fwd 5′- ATGCCATCACTGCCACTCAGA -3’; rev 5′- ACCAGTGGATGCAGGGATGAT -3′)

### 2.9 Statistical analyses

Data are expressed as mean and standard error of the mean. To assess the statistical significance of the presence of seizures, epileptiform spike series and bi-phasic spasm-like events in the CES group, we performed a one sample *t*-test comparing their probability to 0. Presence of any seizures, any spike series or of any ‘spasms’ was assigned a value of 1. Analyses of CRH optical density in amygdala and of CRH-immunoreactive cell numbers in frontoparietal/somatosensory cortex employed Student's *t*-test unless noted otherwise. Significance for all analyses was set at P < 0.05.

## 3 Results

### 3.1 Chronic early-life stress leads to network hyper-excitability and seizures in a subset of rats

Several types of abnormal neuronal network excitability emerged in the CES rats during the days following the stress, and none were observed in the controls. These manifestations of hyper-excitable brain included spike series in amygdala-EEG, electrographic seizures associated with limbic behavioral features (3 of 23 rats), and sudden flexion-type events reminiscent of infantile seizures in humans (11 of 23 rats). In all, these events involved 14 of 23 rats (61%; t_5.85;22_ p < 0.0001; one sample *t*-test).

### 3.2 Abnormal limbic EEGs and limbic seizures arise in a minority of CES rats

**Fig. 1:**
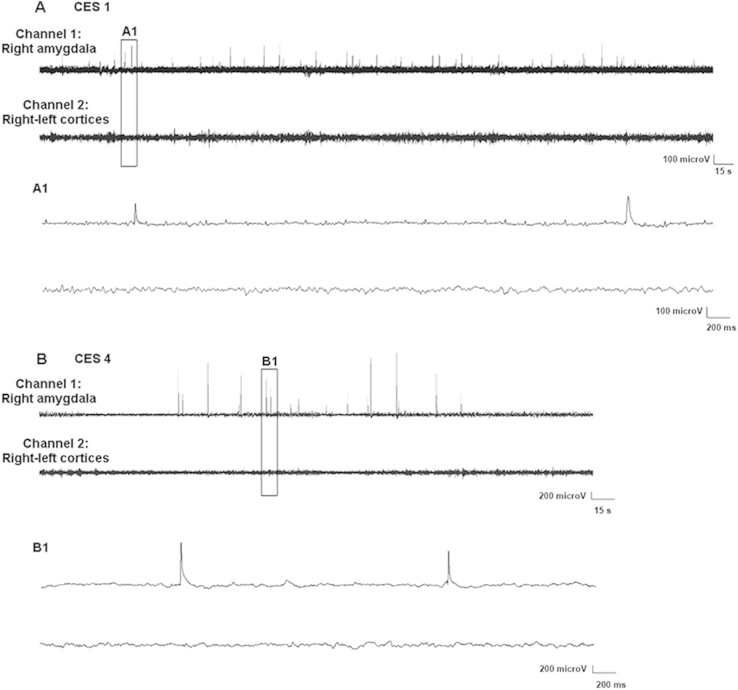
Chronic early-life stress (CES) enhances excitability in amygdala, manifest as the presence of epileptiform spike series. A and B are sample EEGs from two individual rats recorded around two weeks each. Rats were implanted with bipolar electrodes in the right amygdala and with two cortical electrodes, one each over right and left frontoparietal cortices, as described in the methods. The montage used for these animals consisted of: Channel 1: from within the right amygdala (bipolar); Channel 2: Activity between the two cortical electrodes. These epileptiform discharges occurred throughout the 16 and 17 day recordings in the two rats, and their typical contour is shown in an expanded time-scale (of the boxed segments) in A1, B1. Such spike-trains were never observed in the 20 video-EEG recorded control rats.

**Fig. 2:**
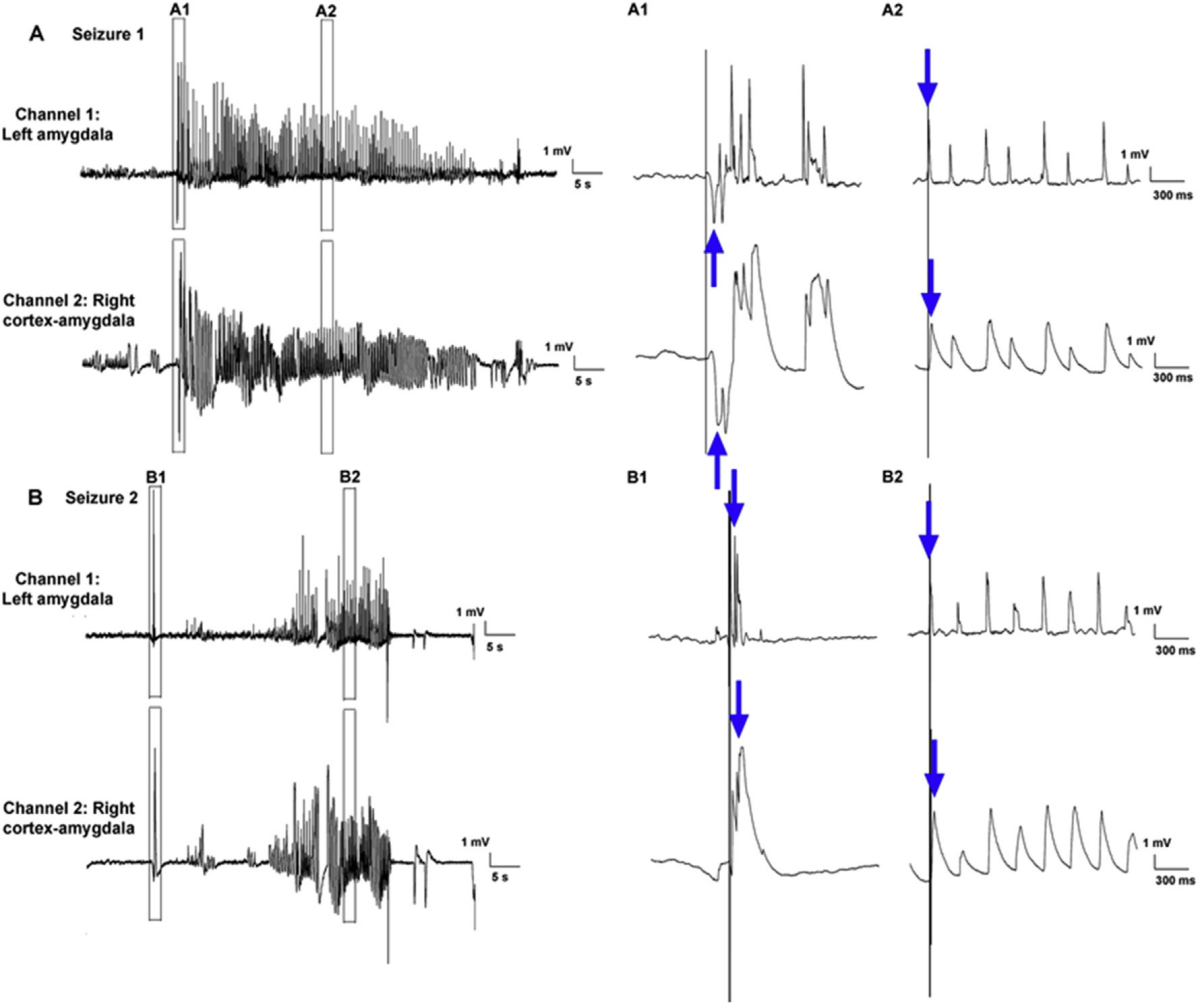
Chronic early-life stress (CES) leads to limbic spontaneous seizures in a minority of rats. Shown are EEGs traces of spontaneous limbic seizures from a CES rat. Rats were implanted bilaterally with bipolar electrodes in amygdala as well as with an electrode over the right frontoparietal cortex. The montage consisted of two channels: Channel 1: intra-left amygdala (bipolar); Channel 2: between one of the twisted electrodes in the right amygdala and the right cortical electrode. A, B show the onset and progression of two spontaneous seizures. The expanded time-scale view (A1,B1) allows observation of the temporal sequence of the onset of the seizures (denoted by the blue arrows). The vertical line aids in discerning that the first voltage deflection associated with the seizure originates in the amygdala channels earlier than in the cortex. The second expanded time-scale views (right) suggest that during the course of the seizure, the amygdala spikes ‘lead’ cortical sharp waves and waves. (For interpretation of the references to color in this figure legend, the reader is referred to the web version of this article.)

Aberrant limbic network activity developed in a subset of rats experiencing CES and in none of the concurrently studied controls. These abnormalities were apparent from the presence of spike series in two of 23 rats (shown in Fig. 1A, B). These spike series were observed during 8 of 16 recording days in one rat, and in 7 of 17 recording days in the other, and were spread throughout the recording period in both. The spike series were apparent only in bipolar intra-amygdala EEGs, suggesting their limbic origin. Frank limbic epileptic events took place in two CES rats and consisted of limbic seizures which were diagnosed on both EEG and on the concurrent videos (Fig. 2). The seizures, lasting 6–70 s, occurred on day 24 or 25 of life, i.e., in weanlings, in which hippocampal development approximates that of young children (Racine, 1972). Specifically, one rat had two seizures 90 s apart (durations: 62 and 41 s) on P25; the seizures arose first in the left amygdala and propagated rapidly to the right amygdala-cortex lead (A1, B1 in Fig. 2). The second rat had one overt seizure (duration: 7 s) on P24, detected in the right amygdala. The same rat had abnormal, epileptiform activity manifest as spike series throughout the monitoring period of P18–P32.

The behavioral manifestations of the seizures were typical for limbic seizures: sudden cessation of activity (Racine stage 0) (Racine, 1972) accompanied by facial automatisms (Racine stage 1) and prolonged immobility with staring. A body jerk signaled the onset and the end of the events. Thus, CES during postnatal days 2–9 led to overt, spontaneous seizures in 2/23 (9%) of subjects and to amygdala seizures and/or spike series in 3/23 (13%). However, the majority of abnormal network hyper-excitability following CES manifested as abnormal age-specific events (in 11/23; rats, 48%) described below.

### 3.3 Chronic early-life stress provokes age-specific epileptic events

**Fig. 3:**
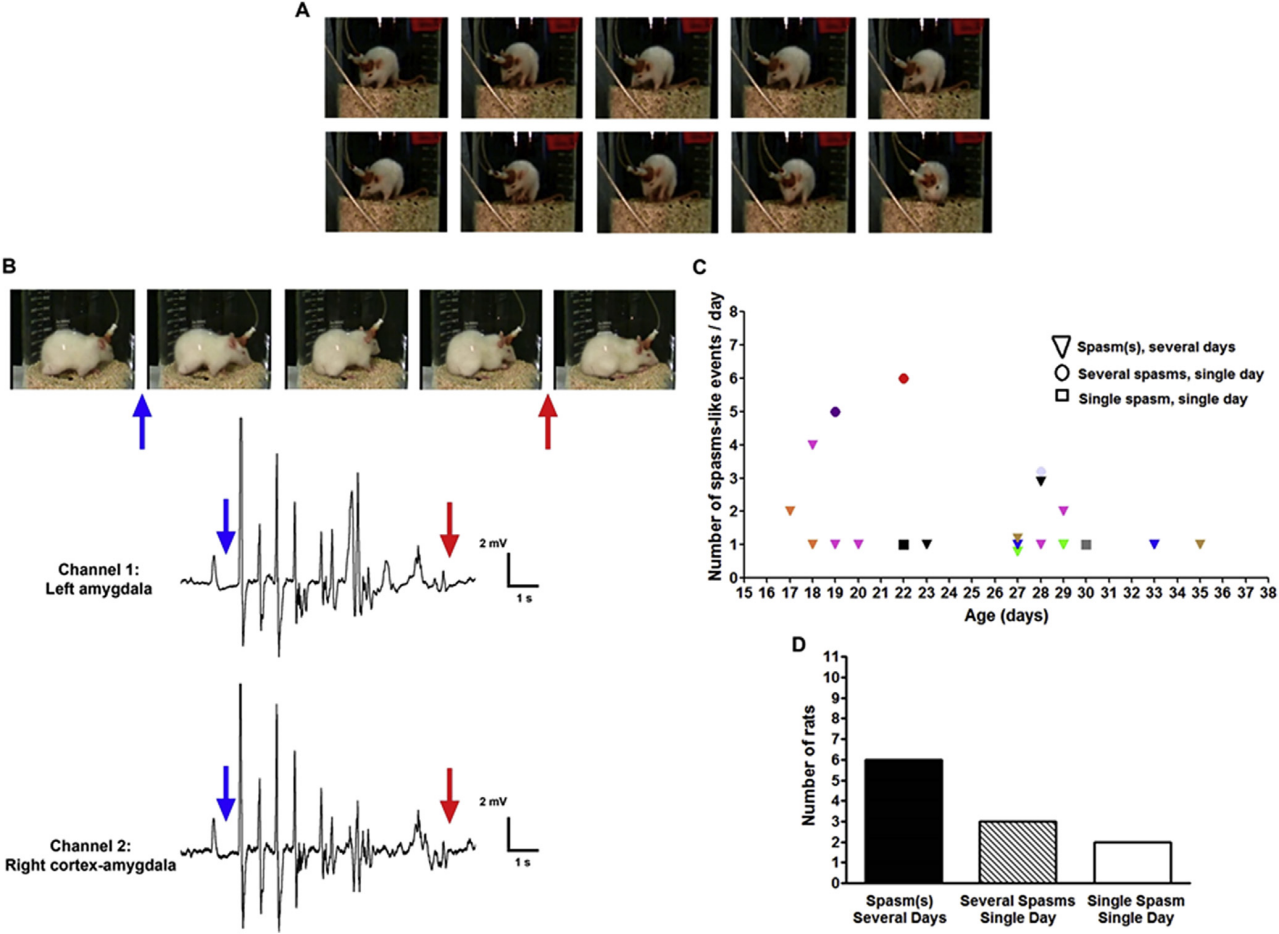
CES provokes flexion events resembling human infantile spasms in 11/23 rats. (A) Serial still images from a video recording of a CES rat cortical electrodes. These images depict the onset and progression of two consecutive spasm-like behaviors: In the event in the top row, note the body flexion of the rat. The sustained, slower phase of this flexion is more apparent in the spasm-like seizure shown in the bottom row. (B) Correlation of spasm-like behaviors and EEG in a different rat, with electrodes within the amygdala. The blue arrow points to the concurrent onset of behavioral flexion and epileptiform discharges. The behavioral event ends at the time noted by the red arrows. (C–D) Temporal and quantitative distribution of spasm-like events among rats. In C, each animal is represented by a symbol and color; the X axis is the age of the rat, in days, and the Y axis is the number of spasms for each days. Triangles denote rats who had multiple spasms over multiple days. These were the majority (6/11), as is depicted in (D). Circles denote rats (n = 3) that had several spasms during a single recording day, and squares denote two rats in whom only a single spasm was captured. Note that rats were recorded for only 1–2 h per day prior to weaning, and thus the actual number of spasms was likely much higher. (D). The majority of rats had flexion, spasm-like events multiple times over several days.

In humans, certain seizure types and epilepsies are strongly age-dependent. Infantile spasms are a severe and relatively common epilepsy syndrome of infancy that responds to stress hormones (Hrachovy et al., 1983, Snead et al., 1989, Baram et al., 1996, Mackay et al., 2004, Stafstrom et al., 2011). In addition, levels of ACTH and cortisol were found to be abnormal in CSF of infants with IS (Baram et al., 1992a). These findings suggested that stress and stress mediators might be involved in the hyper-excitability involved in these types of developmental seizures (Baram, 1993, Huang, 2014, van Campen et al., 2012, Baram and Hatalski, 1998, Kumar et al., 2011). Therefore, we examined for spasm-like events in rats exposed to CES. Bi-phasic, spasm-like events were detected between P17 to 35 in 11 CES rats (48%) (Fig. 3A–C). This number is likely an underestimate, because prior to P21 rats were recorded only for 1–2 h per day. The behavioral manifestations consisted of bi-phasic motions, i.e., rapid flexion of head and body and a slower phase of relaxation (see sequence of two events in Fig. 3A). The EEG accompaniments of these events were typically a spike or series of spikes followed by a short period of reduced voltage, reminiscent of those observed in spasms of human infants. These were found in all 6 rats with amygdala electrodes (Fig. 3B), and in 3 of 5 rats in whom the location of the electrodes was hippocampal or cortical (the technical quality of the remaining 2 precluded critical analysis). The majority of rats (6/11) had several spasm-like events over several days, as shown in Fig. 3C, where color-coded triangles denote each of these rats, and in Fig. 3D. Other rats were observed to have several spasms during a single day, and 2 rats had a single event only during the short time-windows of video and EEG monitoring.

### 3.4 Effects of chronic early-life stress on amygdala expression of CRH

**Fig. 4:**
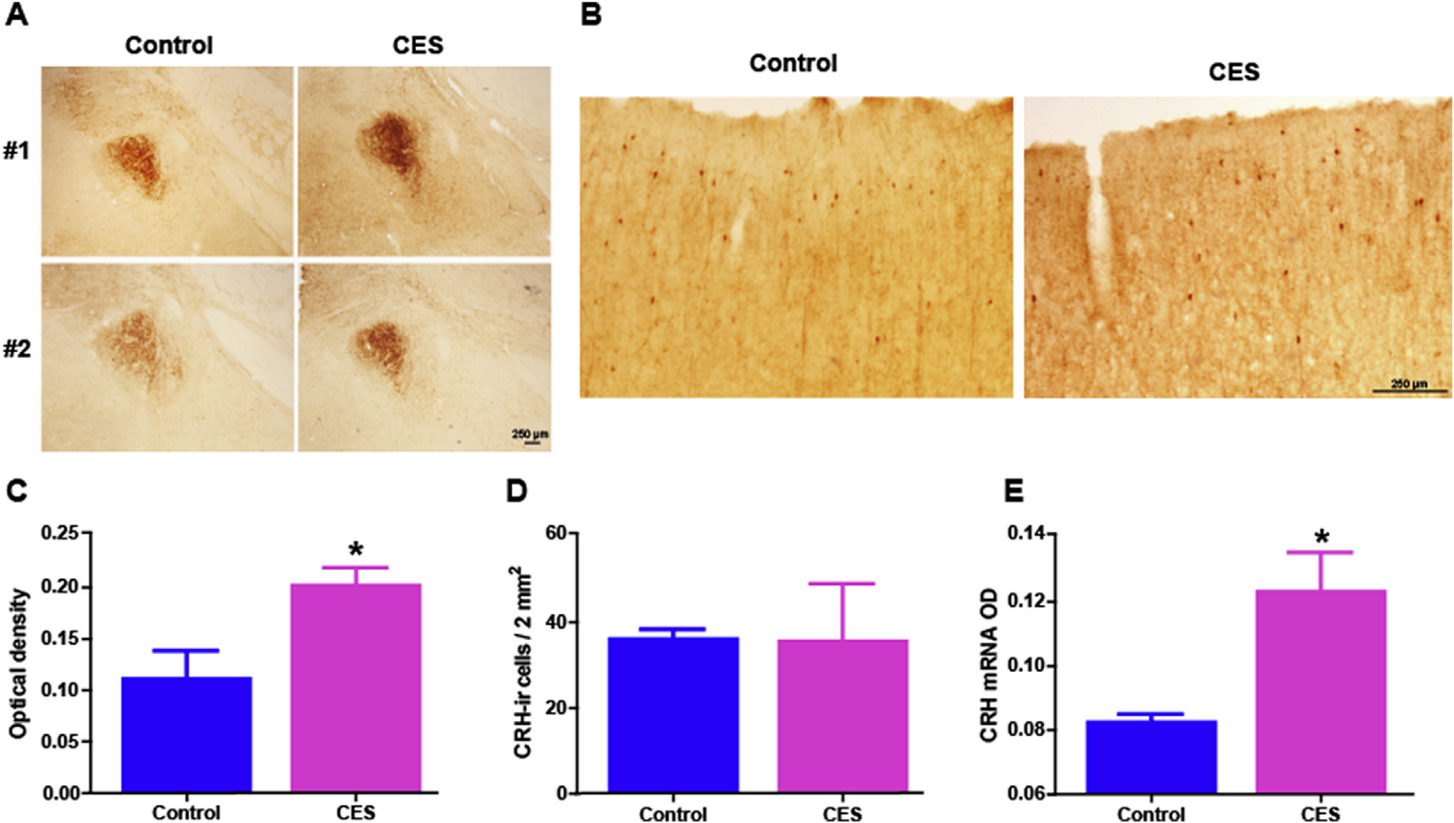
Corticotropin releasing hormone (CRH) expression is augmented in the amygdala of CES rats. (A) Representative photomicrographs of amygdalae after immunohistochemistry using an antiserum directed against CRH. CRH immunoreactivity (ir) was enhanced in the amygdala of CES rats compared with controls at P45 as shown in C: Semi-quantitative analysis of CRH expression levels revealed a significant increase of the optical density of CRH-ir in rats that experienced CES, (*p* = 0.048). (B) In frontoparietal-cortex, CRH-ir cell number and density did not differ significantly in the same control and CES rats distinguished by amygdala expression. Representative micrographs show the typical distribution of CRH-expressing cortical interneurons. (D) A graph depicting the number of cells expressing CRH above detection levels in an area of 2 square mm (controls: 36.3 ± 2.1; CES: 35.8 ± 13.2; p = 0.97, t-test with Welch correction for unequal variance). Cell numbers were not used in amygdala sections because, as apparent in the photos in A, most CRH in this nucleus is found in fibers. (E) At the age of onset of spasm-like events (pre-weaning or infancy, P19), CRH mRNA expression levels were borderline higher in CES rats compared to controls (CES: 0.12 ± 0.01; controls: 0.08 ± 0.002, n = 3–4 per group; *p* = 0.04, Mann–Whitney test). Scale: 250 μm.

The presence of spontaneous ictal events, both spasm-like and limbic seizures in immature rats that had experienced CES indicated intrinsic hyper-excitability of the limbic circuit. In addition, the electrographic data suggested a strong involvement of the amygdala. Indeed, this limbic region has been strongly implicated in a number of developmental seizures in rodent models (Ben-Ari et al., 1984, Baram et al., 1992b, Baram et al., 1997). These findings provided impetus for identifying pro-convulsant, stress-dependent molecules within the amygdala. CRH is an excitatory and pro-convulsant peptide (Aldenhoff et al., 1983, Hollrigel et al., 1998) that provokes amygdala-based limbic seizures in immature rodents (Baram et al., 1992b). In addition, acute or intermittent early-life stress is known to increase CRH expression in a number of brain regions including amygdala (Hatalski and Baram, 1997, Ivy et al., 2010). Therefore, we asked whether CES resulted in augmented CRH expression levels in the amygdala of immature rats. We first estimated CRH mRNA levels in the central amygdala nucleus at the onset of the spasm-like events (P19) using ISH, and found a borderline significant difference between the rats that experienced CES and the control group (0.12 ± 0.01 and 0.08 ± 0.002, *n* = 3–4 per group; *p* = 0.044, Mann–Whitney test; Fig. 4E). qRT-PCR analyses of dissected amygdalae (n = 8 CES, 7 controls) yielded large variances and no conclusive results. Therefore, we looked more directly at peptide expression and examined a somewhat later time-point. Because the majority of amygdala CRH is found in fibers, we employed immunohistochemistry and evaluated optical density of CRH-immunoreactive (ir) signal in carefully matched sections of the central nucleus of amygdala without knowledge of groups. In rats sacrificed on P45, CRH-ir was significantly increased in rats experiencing chronic early-life stress compared to controls (*p* = 0.048; Fig. 4A, C). The augmentation of CRH expression was selective, and was not observed in cortical regions of the same rats. In the cortex (and hippocampus) the number of neurons in a given area that express CRH at levels detectable using immunohistochemistry provides a reliable measure of expression levels of the peptide (Chen et al., 2001, Ivy et al., 2010). Therefore, we counted the numbers of CRH-ir neurons per unit area (Fig. 4B, D) and found no significant differences attributable to CES (per 2 mm^2^, controls: 36.3 ± 2.1; CES: 35.8 ± 13.2; p = 0.97 t-test with Welch correction for unequal variance). Whereas immunohistochemistry may not be fully quantitative, these data suggest that CES augmented the expression of CRH in amygdala and not in cortex in an enduring manner. Elevated peptide levels and release, in turn, should strongly increase network excitability (Baram and Hatalski, 1998, Aldenhoff et al., 1983, Hollrigel et al., 1998).

## 4 Discussion

The studies described here are the first to examine the effects of **chronic** early-postnatal stress on increased excitability in hippocampal-amygdala circuits. They also report on technically challenging daily EEG and video recordings in pre-weanling rats, during developmental periods in the rodent that are parallel to infancy and childhood in humans (Avishai-Eliner et al., 2002). They demonstrate that CES provokes abnormal hyper-excitability in the majority (61%) of developing rats. This hyper-excitability is apparent as EEG spike series, flexion-type seizures and/or limbic seizures. Finally, the findings support the notion that the mechanisms of hyper-excitability might involve augmented levels of the proconvulsant peptide, CRH, in seizure-prone limbic structures including the amygdala.

### 4.1 Studying the consequences of **chronic** early-life stress on excitability in hippocampal-amygdala circuits of ‘infant’ and ‘pre-adolescent’ rodents

Whereas it is difficult to provide objective measures of CES in humans, low socioeconomic status (SES) is commonly considered a surrogate measure. In accord, the prevalence and incidence of epilepsy is higher in resource-poor countries and in children and adults from low SES (Shamansky and Glaser, 1979, (Heaney et al., 2002), but see (Hesdorffer et al., 2005)). Surprisingly, it has been difficult to generate models of CES in neonatal rodents (Molet et al., 2014). Most studies have relied on intermittent or acute maternal separation that, in turn, provokes intermittent or acute stress in pups. Here we employed a paradigm of chronic stress that lasts for a week and is characterized by persistently augmented stress-hormone levels and even adrenal hypertrophy, a hallmark of chronic stress (Gilles et al., 1996, Avishai-Eliner et al., 2001, Ivy et al., 2008). The continuous stress in pups derives from a fragmented and unpredictable maternal care, induced by simulation of low SES in the rodent cages by limiting nesting and bedding available to the dam (Ivy et al., 2008, Baram et al., 2012). This provokes stress in the dam (Molet et al., 2014) and alters her nurturing behaviors (Molet et al., 2014). This paradigm enables probing the consequences of *bone fide****chronic*** early-life stress on brain excitability, seizures and epilepsy. We also successfully recorded EEG chronically in pre-weanling rats, a technical challenge because of potential inanition or maternal cannibalism. These combined advances enabled us to detect EEG markers of network hyper-excitability (Staley et al., 2011) and seizures in the majority of CES rats already during development.

The possibility might be considered, that a “second hit” comprised of being tethered to the EEG and being monitored might lead to epilepsy. As we have not found this in control rats, the remaining fined argument is that the second hit might provoke epilepsy only in stress-compromised rats but not in controls. The stress of the theoretical second hit might be divided into 2 components: the stress of surgery and the stress of video-monitoring. We have measured stress hormones in implanted rodents, and by days later, corticosterone levels were similar to those of un-implanted controls (Chen et al., 2006). We also reported that the early-life stress does not appear to increase the neuroendocrine response to a second stress in either adolescent (Molet et al., 2013) or adult (Brunson et al., 2005) rats. The remote possibility that the merely being tethered might provoke epilepsy in prior-stressed rodents cannot be fully excluded.

### 4.2 Typical seizures arise in a minority of immature CES rats

The consequences of early-life stress on epileptogenesis have been a topic of intense interest (Joëls, 2009, Huang, 2014). In the majority of experimental approaches in rodents, pre-, peri- or postnatal stresses or glucocorticoids were applied to dams and pups were tested for their susceptibility to chemical convulsants or kindling (Edwards et al., 2002, Salzberg et al., 2007, Desgent et al., 2012, Schridde et al., 2006, Kumar et al., 2011). To our knowledge, the current study is the first to demonstrate that CES leads directly to spontaneous epileptic events including seizures without a second hit. The reason for the low number of typical spontaneous limbic seizures in our cohort is unclear. The low detection rate might derive from intermittent recordings in pre-weanling rats. It is also possible that limbic seizures are not typical in immature rodents, and more epileptic phenomena are flexion-spasm like events associated with epileptiform EEGs. Indeed, flexion-type seizures have been reported in immature rats challenged with NMDA (Velísek et al., 2007, Chachua et al., 2011, Mares and Velísek, 1992); hyperthermia (Baram et al., 1997), a triad of brain insults (Scantlebury et al., 2010) or genetic mutations (Price et al., 2009, Marsh et al., 2009). Not surprisingly, these types of events constituted the majority of ictal events observed here.

It is unlikely that genetic or traumatic factors provoked the hyper-excitability reported here. We excluded the presence of brain injury by examining brains postmortem. In addition, the rat strain used has no known spontaneous seizures or EEG abnormalities (though some have reported absence-like events later in life). Over the years, we have recorded EEGs and videos from over 100 control rats and found no abnormalities.

### 4.3 Flexion seizures in immature CES rats resemble human infantile spasms

The appearance of seizures in humans and rodents is age-specific, and this might result from immature stages of development that promote seizure propagation in certain circuits and prevent their propagation via other, less mature networks (Marsh et al., 2009, Holmes and Ben-Ari, 1998, Dulac et al., 2013, Baram, 2012). The age-specific epilepsy, infantile spasms, is characterized by rapid flexion of the head and torso, followed by a longer persistent flexion and relaxation (Hrachovy et al., 1983, Snead et al., 1989, Baram et al., 1996, Stafstrom et al., 2011, Nordli, 2002). In a number of genetic and pharmacological immature rodent models, as well as the CES model described here, similar flexion seizures have been generated (Velísek et al., 2007, Mares and Velísek, 1992, Price et al., 2009, Marsh et al., 2009, Galvan et al., 2000, Lee et al., 2008, Cortez et al., 2009). It has been far more difficult to generate in rodents the typical background EEG found in infants with IS. This is likely a result of dichotomous cortical development stages in humans and rodents (Avishai-Eliner et al., 2002, Holmes and Ben-Ari, 1998, Baram, 2012), as well as the absence of sulci and gyri in the latter. To our knowledge, whereas a number of infantile spasm models have been generally accepted, chaotic, hypsarrhythmia-like pattern has been detected only in one (Lee et al., 2008).

Here, as in the majority of established rodent models, we recapitulate the behavioral manifestations of the seizures and find accompanying epileptiform discharges. We do not propose that the events provide an optimal model for IS: this is a controversial topic with differing opinions among experts (Stafstrom et al., 2011, Stafstrom and Holmes, 2002). We simply demonstrate that CES provokes hyper-excitability that results in the emergence of typical developmental spasm-like events.

### 4.4 Stress hormones as potential mechanisms of hyper-excitability and epilepsy after CES

How might CES increase brain excitability? Stress effects on the brain involve the canonic hypothalamic pituitary adrenal (HPA) axis, as well as a number of networks including a limbic-neuroendocrine circuit (McEwen, 2007, Baram and Hatalski, 1998, Joëls and Baram, 2009). The effects of stress on the brain are mediated by several types of molecules, including neurotransmitters, peptides and steroid hormones, which are candidates for mediating the influence of CES on network excitability. Corticosteroid hormones act via two nuclear receptor types (Joëls and Baram, 2009), mineralocorticoid receptor (MR) that is highly expressed in limbic areas such as the hippocampus; and the glucocorticoid receptor (GR) ubiquitously expressed in the brain and enriched in the hippocampus. GR and MR activation can influence neuronal excitability through rapid nongenomic pathways (Joëls, 2009, Joëls and Baram, 2009), as well as via delayed, transcriptional regulation of the expression of hundreds of genes (Joëls, 2009, McEwen, 2007). Functionally, GRs tend to normalize excitability that was raised during the initial stage of the stress response. Thus, in the context of stress, steroids augment excitability acutely, but their long-lasting effects are less clear.

The neuropeptide CRH may also mediate the effects of stress on excitability in the developing brain. Stress activates expression and release of CRH from CRH-expressing neurons in several limbic regions including hippocampus and amygdala (Roozendaal et al., 2002). The peptide increases the firing of CA1 pyramidal neurons in both mature and developing hippocampus, (Aldenhoff et al., 1983, Hollrigel et al., 1998) and provokes limbic seizures that seem to commence in amygdala in immature rodents (Baram et al., 1992b). Activation and augmentation of CRH expression might take place during the numerous developmental insults that commonly precede human developmental epilepsies including IS. Rodent models suggest that treatment with ACTH directly suppresses amygdala CRH expression via melanocortin receptors (Brunson et al., 2001a, Hatalski et al., 1998, Snead, 2001). In infants, ACTH at high doses is generally more effective in suppressing IS and the abnormal EEG associated with them, as compared to maximal doses of corticosteroids (Snead et al., 1989, Baram et al., 1996, Mackay et al., 2004, Stafstrom et al., 2011), and the additional ACTH efficacy might derive from direct effects on amygdala CRH. Thus, the current findings suggest that the mechanism of action of ACTH and corticosteroids in IS via their actions on the stress system rather than via other potential mechanisms such as anti-inflammatory effects (Tekgul et al., 2006), (Dedeurwaerdere et al., 2012).

### 4.5 Clinical relevance and implications

Infants and children with IS or epilepsy have evidence of abnormal stress hormone levels in the CSF (Baram et al., 1992a, Baram et al., 1992b) and cortex (Wang et al., 2001). These facts, coupled with the universal response of some developmental seizures to stress hormones implicate stress as a contributor to early-life brain hyper-excitability, seizures and epilepsy. The current studies demonstrate that CES directly enhances brain excitability. Thus, CES might provoke frank epileptogenesis in a minority of affected infants and children, and contribute to vulnerability to seizures and epilepsy in a larger proportion. Because stress is largely unavoidable, uncovering the underlying mechanisms is vital for developing preventive interventions.

## Conflicts of interest

The research was supported by the National Institute of Health, RO1 NS28912, P50 MH096889.

Equipment (EEG, Telemetry, analysis software) used in the study was purchased using a gift from Questcor, Inc., received in 2009.
